# UTOPIA—User-Friendly Tools for Operating Informatics Applications

**DOI:** 10.1002/cfg.359

**Published:** 2004-02

**Authors:** S. R. Pettifer, J. R. Sinnott, T. K. Attwood

**Affiliations:** 1 Department of Computer Science University of Manchester Oxford Road Manchester M13 9PL UK; 2 School of Biological Sciences University of Manchester Oxford Road Manchester M13 9PL UK

## Abstract

Bioinformaticians routinely analyse vast amounts of information held both in large remote databases and in flat data files hosted on local machines. The contemporary toolkit available for this purpose consists of an *ad hoc* collection of data manipulation tools, scripting languages and visualization systems; these must often be combined in complex and bespoke ways, the result frequently being an unwieldy artefact capable of one specific task, which cannot easily be exploited or extended by other practitioners. Owing to the sizes of current databases and the scale of the analyses necessary, routine bioinformatics tasks are often automated, but many still require the unique experience and intuition of human researchers: this requires tools that support real-time interaction with complex datasets. Many existing tools have poor user interfaces and limited real-time performance when applied to realistically large datasets; much of the user's cognitive capacity is therefore focused on controlling the tool rather than on performing the research. The UTOPIA project is addressing some of these issues by building reusable software components that can be combined to make useful applications in the field of bioinformatics. Expertise in the fields of human computer interaction, high-performance rendering, and distributed systems is being guided by bioinformaticians and end-user biologists to create a toolkit that is both architecturally sound from a computing point of view, and directly addresses end-user and application-developer requirements.


**Comparative and Functional Genomics**
*Comp Funct Genom* 2004; **5**: 56–60.
Published online in Wiley InterScience (www.interscience.wiley.com). **DOI:** 10.1002/cfg.359

Conference Review

### UTOPIA — user-friendly tools for operating informatics applications

S. R. Pettifer[1]*, J. R. Sinnott[1] and T. K. Attwood[1,2]
[1] *Department of Computer Science, University of Manchester, Oxford Road, Manchester M13 9PL, UK*
[2] *School of Biological Sciences, University of Manchester, Oxford Road, Manchester M13 9PL, UK*

**Correspondence to: S. R. Pettifer, Department of Computer Science, University of Manchester, Oxford Road, Manchester M13 9PL, UK. E-mail: srp@cs.man.ac.uk*

Received: 18 November 2003
Revised: 20 November 2003
Accepted: 20 November 2003

### Abstract

**Bioinformaticians routinely analyse vast amounts of information held both in large remote databases and in flat data files hosted on local machines. The contemporary toolkit available for this purpose consists of an *ad hoc* collection of data manipulation tools, scripting languages and visualization systems; these must often be combined in complex and bespoke ways, the result frequently being an unwieldy artefact capable of one specific task, which cannot easily be exploited or extended by other practitioners. Owing to the sizes of current databases and the scale of the analyses necessary, routine bioinformatics tasks are often automated, but many still require the unique experience and intuition of human researchers: this requires tools that support real-time interaction with complex datasets. Many existing tools have poor user interfaces and limited real-time performance when applied to realistically large datasets; much of the user's cognitive capacity is therefore focused on controlling the tool rather than on performing the research. The UTOPIA project is addressing some of these issues by building reusable software components that can be combined to make useful applications in the field of bioinformatics. Expertise in the fields of human computer interaction, high-performance rendering, and distributed systems is being guided by bioinformaticians and end-user biologists to create a toolkit that is both architecturally sound from a computing point of view, and directly addresses end-user and application-developer requirements. Copyright © 2004 John Wiley & Sons, Ltd.**

**Keywords:** **sequence analysis; visualization; human computer interaction**

### Introduction

A common tool of the bioinformatician's trade is the sequence alignment, e.g. in searching a database with an unknown query sequence, programs such as FastA [1] and BLAST [2] generate pairwise alignments between the query and target sequences in the database. The best-scoring matches are reported, and the user then examines the resulting alignments to determine the biological significance of the hits. If a group of related sequences is identified at the top of a hit-list, it is usual then to create a multiple alignment, in order to be able to visualize the most conserved regions (motifs) of the family, which may be indicative of particular structural or functional features. More sensitive database searches may then be performed using just these conserved motifs, allowing more distant family members to be retrieved and analysed. If a three-dimensional structure of a member of the family is known, the next step might involve alignment of the query with the sequence of known structure, and subsequently pinpointing conserved residues within the protein fold. This might give clues as to the whereabouts of, say, molecular interaction or binding sites, so shedding light on possible aspects of the unknown protein's functionality. Another form of analysis might involve the construction of phylogenetic trees from multiply aligned family members, thereby helping

Copyright © 2004 John Wiley & Sons, Ltd.

to elucidate their evolutionary relationships and, again, potentially facilitating functional characterization of the unknown protein.

Today, such tasks are, in principle, relatively straightforward to perform. In practice, however, they usually involve the use of diverse tools and databases, of which some are stored locally, while others must be accessed remotely via Telnet or the Internet. By way of illustration:

- Some require interaction with Web forms, and subsequent retrieval of information from poorly-structured HTML pages, e.g. BLAST.
- Some involve interaction with applets, and retrieval of results via e-mail, e.g. CINEMA [3].
- Others require use of applications available on the user's PC, or on local Unix/Linux-based servers, e.g. CLUSTALW [4], PHYLIP [5].
- Still others might require remote log-in to resource centres such as HGMP-RC, and subsequent file-transfer between remote and local machines, e.g. GCG [6].

There are several reasons why this diversity of approaches has arisen: (i) many users still don't know (and don't want to have to find out) how much they can do via the Internet (they are comfortable with a self-contained, desk-top package, supplemented with an occasional BLAST search); (ii) some users are not allowed to make extensive use of the Internet (e.g. industrialists), so must have resources and tools available on platforms in-house; (iii) many bioinformatics tools have only been written as Unix/Linux-based applications and have not been ported to Windows or MacOs environments (which are the worlds inhabited by most biologists); (iv) other tools have only been written as applets, ostensibly to obviate portability problems; (v) most packages come bundled with tools and databases that date quickly, making access to the latest algorithms and data via the Internet still essential; and (vi) some packages and databases have prohibitively expensive or restrictive licensing arrangements, and are therefore only feasibly accessible at remote multi-user resource centres. Surprisingly then, there is no current solution or working environment that makes access to bioinformatics databases and tools as easy as it could or should be.

Turning our attention away from the working environment itself to focus on the types of analysis package available for biologists and bioinformaticians, we encounter still more problems. To return to our example of sequence alignments, we find that the programs available range from: (i) stand-alone automatic multiple alignment tools, accessible as command-line driven applications or via Web forms, e.g. CLUSTALW; (ii) components of large (often commercial) integrated packages, e.g. pileup in GCG; (iii) command-line driven manual alignment editors with X-windows interfaces, e.g. XALIGN [7]; (iv) manual editors written in Java as applets e.g. CINEMA, JalView [8]; or (v) X-windows-based alignment viewers, e.g. Sonnhammer's BelVu. The multiplicity of tools is not merely confusing for the bench biologist, it is wasteful for both user and developer, because most have not been developed in a reusable manner. To give a trivial example, virtually all of these programs use different input–output formats (e.g. NBRF-PIR, FastA, CLUSTAL, GDE, PHYLIP, MSF, to name but a few). Thus, if a user wishes to export an alignment from an automatic package into a manual editor, he/she must first use a program to convert between formats. Similarly, if a developer wants to integrate an automatic alignment tool into an existing manual editor (or vice versa), then he/she must also write, or bundle, an appropriate format-exchange program into the system.

### The challenge

In light of these issues, we felt that a new perspective was needed on the problem of providing bioinformatics tools and databases in a user-friendly environment. Crucially, we now live in a 'post-genome' data-rich world, in which it is clear that human interaction and visualization techniques are of paramount importance if we are to make further progress. We need systems in which the abilities of the user are supported rather than confounded by computational tools, where the user does not feel intimidated by cumbersome or inappropriate interfaces, does not have to worry about underlying file-types or operating systems, but can use whatever tools are needed within a clear, visually supportive and intuitive, Grid-compatible framework. Within such a system, we need, for example, to be able to: (a) align sequences (manually and/or automatically), whether protein, DNA

Copyright © 2004 John Wiley & Sons, Ltd. 

or RNA; (b) search databases, whether sequence-, motif-, structure-, mutation-, literature-based, etc; and (c) visualize, and interact with, 2D and 3D representations, whether of molecular structures, protein–protein interactions, phylogenetic trees, dot-plots/surfaces, gene-expression data, etc. The environment needs to offer different views of the user's workspace, e.g. via a resource browser that indicates the locations, types, sizes and ages not only of databases, but also of input and output files (sequences, structures, alignments, BLAST results, or whatever). The system needs to be customizable, so that databases and tools can be updated automatically via appropriate agent software, either without troubling the user or by notifying him/her that new versions of various resources are now available for installation. And the environment also needs to be collaborative, allowing users in different locations to visualize and interact with the same data — this would be especially useful in projects based in different geographical locations, or in training/community-learning settings.

### The problem

The difficulties that bioinformaticians encounter can be traced to the following problems:

1. *Infrastructure*: the underlying data being manipulated are semantically complex and stored in a distributed heterogeneous manner. The current infrastructure (primarily the Web, or low-level Internet protocols and tools) means that this complexity is continuously exposed to the user in unhelpful ways, creating significant barriers to actual data analysis and interpretation.
2. *User tools/applications*: existing visualization/analysis tools and applications are built on the current low-level infrastructure using an *ad hoc* collection of general-purpose programming techniques and interfaces. They have nothing approaching the coherence and integration of, say, the typical 'office software suite' and yet aim to work with significantly more complex datasets than are represented by a collection of word processor documents and spreadsheets.

Our aim is to tackle these related problems together in order to produce a coherent solution — UTOPIA!

### The UTOPIA system

The design of the UTOPIA system is guided by three principles:

1. *Intuitive interaction.* End-user applications, such as the sequence alignment tool, must be based on established human computer interaction techniques, e.g. the 'desktop paradigm', with its associated files, folders, drag-and-drop and cut-and-paste metaphors, is familiar to the majority of computer users and, applied sympathetically and consistently, is as suitable for protein sequence alignment and structure analysis as it is for word processing and project management.
2. *Reusable components.* Applications should not be monolithic structures, but rather constructed from flexible and extensible open-source software components that can be adapted by other programmers.
3. *Lightweight deployment.* Installation of the system must be as simple and robust as possible, keeping extraneous dependencies on other packages to a minimum.

UTOPIA has three main packages, targeted at different kinds of end user:

1. *Workbench.* This consists of a suite of interworking 'end-user applications' suitable for use by biologists who do not wish to know what is going on beyond the desktop. Available as pre-compiled binaries for Linux, Windows and MacOS X, the workbench package includes the CINEMA sequence alignment tool (see Figure 1), a highly optimized 3D-structure viewer capable of rendering very large molecules in real-time, and a number of other analysis tools, all of which are capable of sharing data and of interacting with remote resources in intuitive ways. Requiring a minimum investment to install, and while being usable tools in their own right, the components of the workbench alone are a form of 'UTOPIA Lite', requiring installation of the UTOPIA Server package for full benefit.
2. *Server.* For users able to invest more effort in the installation of a server, this package provides extended Grid facilities and uniform access to heterogeneous resources via a networked 'virtual file system' (the 'UTOPIA File System' or

Copyright © 2004 John Wiley & Sons, Ltd. 

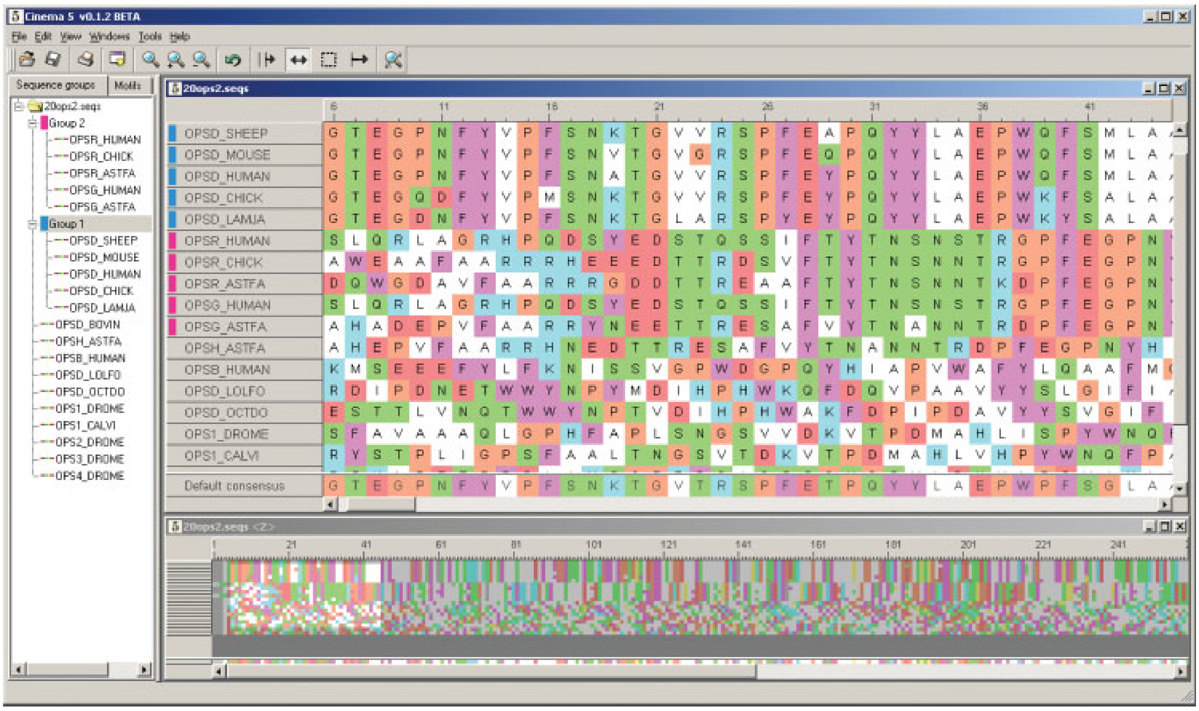

**Figure 1.** Screen shot illustrating one of the core UTOPIA applications — the sequence alignment editor, CINEMA. At the bottom of the screen, an overview illustrates the full extent of the alignment; at the top, the panel illustrates a detail from the N-terminal portion of the alignment. Any number of alignment views may be invoked and manipulated in a given analysis session. The left-hand 'tree' lists the aligned sequences by ID code and may be used to group sequences into closely related families to reduce repetitive operations on each family member. Cut-and-paste, drag-and-drop-type operations work much as would be expected in a drawing or word processing package, enabling straightforward interoperation with other desktop tools

UFS). The software resides on a Unix server, and provides the facility to make Grid and Web resources, such as databases or automated alignment services, appear as files integrated seamlessly with other desktop resources. By tracking the use of these 'virtual files', the UFS server creates metadata and stores provenance information, making event notification possible. For example, the results from a BLAST query are aligned manually using UTOPIA tools, and from this process a number of candidate motifs are identified and submitted to another database. Some time later, the originating database from which the BLAST sequences were drawn is updated to correct an error, with the consequence that the motifs now require revision. On receiving an event from the originating database informing it of this update, the UFS server is able to spot this chain of dependencies between remote resources that are — as far as the user is concerned — simply regular local files, and to initiate a dialogue with the user, perhaps showing a virtual folder containing the now tainted motifs. Since UFS makes remote data appear as regular files, third-party or legacy applications that know nothing of the UTOPIA infrastructure also benefit from its resource management.

3. *Development kit*. For programmers, UTOPIA provides a suite of widgets, ranging from low-level 'Grid-friendly' file requestors and resource browsers to higher level 3D-structure viewing panes and sequence alignment widgets. At all levels of the developer's kit, object-orientated techniques are used to expose the functionality in a coherent manner, allowing the widgets to be embedded and extended straightforwardly. The applications of the workbench package serve as a guide on how to assemble these to make full end-user applications.

Copyright © 2004 John Wiley & Sons, Ltd. 

### Current status

Ultimately, we hope that UTOPIA will provide a basis for consistent and coherent interactive applications, significantly reducing the amount of unnecessary technical detail presented to the working biologist. CINEMA5, the first component of the workbench, is currently available for free download from **http://aig.cs.man.ac.uk/utopia**, with its components and infrastructure to follow.

### Acknowledgements

We are grateful for support for this work from the North West E-Science Centre, the DTI, EPSRC, EMBNet and Sun Microsystems.

### References

1. Lipman DJ, Pearson WR. 1985. Rapid and sensitive protein similarity searches. *Science* **227**: 1435–1441.
2. Altschul SF, Gish W, Miller W, Myers EW, Lipman DJ. 1990. Basic local alignment search tool. *J Mol Biol* **215**(3): 403–410.
3. Parry-Smith DJ, Payne AWR, Michie AD, Attwood TK 1998. CINEMA — a novel Colour INteractive Editor for Multiple Alignments. *Gene* **221**: GC57–63.
4. Thompson JD, Higgins DG, Gibson TJ. 1994. CLUSTAL W: improving the sensitivity of progressive multiple sequence alignment through sequence weighting, position-specific gap penalties and weight matrix choice. *Nucleic Acids Res* **22**(22): 4673–4680.
5. Felsenstein J. 1989. PHYLIP — Phylogeny Inference Package. *Cladistics* **5**: 164–166.
6. Devereux J, Haeberli P, Smithies O. 1984. A comprehensive set of sequence analysis programs for the VAX. *Nucleic Acids Res* **12**(1, pt 1): 387–395.
7. Perkins DN, Attwood TK. 1995. VISTAS — a package for VIsualising STructures And Sequences of proteins. *J Mol Graph* **13**: 73–75.
8. Clamp M, Cuff J, Barton G. 1998. JalView — analysis and manipulation of multiple sequence alignments. *EMBnet News* **5**(4): 16–21.

Copyright © 2004 John Wiley & Sons, Ltd. 
